# Impact of Endogenous Pneumococcal Hydrogen Peroxide on the Activity and Release of Pneumolysin

**DOI:** 10.3390/toxins15100593

**Published:** 2023-09-30

**Authors:** Jasmin Bazant, Benjamin Ott, Martina Hudel, Torsten Hain, Rudolf Lucas, Mobarak Abu Mraheil

**Affiliations:** 1 Institute of Medical Microbiology, German Center for Infection Research, Partner Site Giessen-Marburg-Langen, Justus-Liebig University Giessen, Schubertstrasse 81, 35392 Giessen, Germany; jasmin.bazant@mikrobio.med.uni-giessen.de (J.B.); benjamin.ott@mikrobio.med.uni-giessen.de (B.O.); martina.hudel@mikrobio.med.uni-giessen.de (M.H.); torsten.hain@mikrobio.med.uni-giessen.de (T.H.); 2 Vascular Biology Center, Department of Pharmacology and Toxicology and Division of Pulmonary Critical Care Medicine, Medical College of Georgia at Augusta University, Augusta, GA 30912, USA; rlucas@augusta.edu

**Keywords:** *Streptococcus pneumoniae*, pneumolysin, H_2_O_2_

## Abstract

*Streptococcus pneumoniae* is the leading cause of community-acquired pneumonia. The pore-forming cholesterol-dependent cytolysin (CDC) pneumolysin (PLY) and the physiological metabolite hydrogen peroxide (H_2_O_2_) can greatly increase the virulence of pneumococci. Although most studies have focused on the contribution of both virulence factors to the course of pneumococcal infection, it is unknown whether or how H_2_O_2_ can affect PLY activity. Of note, *S. pneumoniae* exploits endogenous H_2_O_2_ as an intracellular signalling molecule to modulate the activity of several proteins. Here, we demonstrate that H_2_O_2_ negatively affects the haemolytic activity of PLY in a concentration-dependent manner. Prevention of cysteine-dependent sulfenylation upon substitution of the unique and highly conserved cysteine residue to serine in PLY significantly reduces the toxin’s susceptibility to H_2_O_2_ treatment and completely abolishes the ability of DTT to activate PLY. We also detect a clear gradual correlation between endogenous H_2_O_2_ generation and PLY release, with decreased H_2_O_2_ production causing a decline in the release of PLY. Comparative transcriptome sequencing analysis of the wild-type *S. pneumoniae* strain and three mutants impaired in H_2_O_2_ production indicates enhanced expression of several genes involved in peptidoglycan (PG) synthesis and in the production of choline-binding proteins (CPBs). One explanation for the impact of H_2_O_2_ on PLY release is the observed upregulation of the PG bridge formation alanyltransferases MurM and MurN, which evidentially negatively affect the PLY release. Our findings shed light on the significance of endogenous pneumococcal H_2_O_2_ in controlling PLY activity and release.

## 1. Introduction

*Streptococcus pneumoniae* (the pneumococcus) is a commensal colonizer of the human upper respiratory tract in healthy individuals. Epidemiological studies suggest that <10% of adults and up to 27–65% of children are carriers of *S. pneumoniae* [1,2]. *S. pneumoniae* is a major human pathogen that causes invasive (pneumonia, meningitis, bacteraemia) and non-invasive (acute otitis media, sinusitis) diseases. The transmission of *S. pneumoniae* to the lower respiratory tract, the bloodstream, or the meninges can lead to severe illness [3]. Since 2017, the WHO has considered *S. pneumoniae* one of the twelve priority pathogens. *S. pneumoniae* bacteria release the cholesterol-dependent cytolysin (CDC) pneumolysin (PLY), a major virulence factor that can form pores in the host-cell membrane that consist of up to 44 PLY monomers and can reach a diameter of 26 nm [4,5]. In contrast to other CDCs, PLY lacks an *N*-terminal secretion signal sequence that enables an active secretion of PLY [6,7]. Initially, it was thought that PLY is only released from *S. pneumoniae* upon autolysis by autolysin LytA or following antibiotic treatment [8,9]. Later, it turned out that PLY release also takes place during early growth phases before activation of the autolytic cascade [10]. Furthermore, PLY release occurs also in the absence of the major pneumococcal autolysin, LytA, an N-acetyl-muramoyl-L-alanyl amidase [11]. Released pneumococcal PLY induces intracellular Ca^2+^ influx and cell lysis, as well as modulating host immune response [12,13]. Additionally, due to its catalase negativity, *S. pneumoniae* generates high levels of hydrogen peroxide (H_2_O_2_) by the activity of pyruvate oxidase (SpxB) and lactate oxidase (LctO) [14,15]. Such high H_2_O_2_ levels secreted by *S. pneumoniae* in the alveolar space suppress ENaC-α transcription and can inhibit the Na^+^–K^+^ pump. As a consequence, vectorial Na^+^ transport is impaired and alveolar flooding can occur, which can precipitate a lethal hypoxaemia by impairing gas exchange. Another consequence of *S. pneumoniae* H_2_O_2_ release is an increased production of mitochondrial ROS in the host cells, as observed during infection with wild-type *S. pneumoniae* and with a pneumolysin-negative mutant strain, but not with a SpxB-negative *S. pneumoniae* strain (reviewed in [15]). Additionally, *S. pneumoniae* exploits the bactericidal effects of H_2_O_2_ to reduce nasopharyngeal colonization by eliminating microbial competitors like *Staphylococcus aureus*, *Haemophilus influenzae*, and *Neisseria meningitidis* [16,17,18]. However, *S. pneumoniae* possesses several proteins that are involved in protection against oxidative stress and confer resistance to endogenously generated H_2_O_2_ [19]. Furthermore, although SpxB is responsible for endogenous H_2_O_2_ production, it has been found to be required for survival during exposure to high levels of exogenously added H_2_O_2_ [20]. In addition to harmful effects on other bacteria, the pneumococcus utilizes H_2_O_2_ as a virulence determinant. Released pneumococcal H_2_O_2_ diffuses through the host-cell membrane and induces toxic effects based on DNA double-strand breaks, which precede apoptosis [21]. H_2_O_2_ is also involved in the suppression of inflammasome-dependent innate immunity and thereby facilitates bacterial colonization in the host [22]. Additionally, *S. pneumoniae*-derived H_2_O_2_ was found to be able to elevate mitochondrial reactive oxygen species (mtROS) and to initiate the endoplasmic reticulum (ER) stress response via the PERK pathway during infection [23]. H_2_O_2_ was also found to improve pneumococcal intracellular survival by compromising the degradative capacity of the host lysosomes in brain microvascular endothelial cells [24].

During its growth within the lungs, *S. pneumoniae* releases H_2_O_2_ and PLY. Both virulence factors cause significant DNA damage and apoptosis in both type 1 and type 2 human alveolar epithelial cells [21,22,23,24,25], which are responsible for alveolar fluid clearance [26]. Furthermore, PLY and H_2_O_2_ induce epithelial and endothelial barrier dysfunction in the lungs during infection and thereby promote the spread of *S. pneumoniae* [27,28,29]. Notably, *S. pneumoniae* infection in human lung tissue disrupts tight junctions at both the epithelial and endothelial barriers and causes oxidative stress [30,31]. Although the contribution of PLY and H_2_O_2_ to the virulence of *S. pneumoniae* has been the subject of several investigations, little is known about a possible interaction between both virulence factors.

In this study, we examined the ability of H_2_O_2_ generated by *S. pneumoniae* to affect the haemolytic activity of PLY. Our results demonstrate that H_2_O_2_ negatively affects the haemolytic activity of PLY in a concentration-dependent manner. The substitution of the unique and highly conserved cysteine residue in PLY diminishes the negative effect of H_2_O_2_ on PLY activity. Additionally, we found that the supernatants of *S. pneumoniae* mutants impaired in their H_2_O_2_ production due to deletion of *lctO*, *spxB*, or both are less haemolytic. The reduced haemolytic activity of the supernatants was found to be due to the gradually decreased release of PLY. We identified increased expression of a set of cell-wall proteins and choline-binding proteins as possible reasons for the H_2_O_2_-dependent decrease in PLY release.

## 2. Results

### 2.1. H_2_O_2_ Affects the Haemolytic Activity of PLY 

During its growth, *S. pneumoniae* generates and releases H_2_O_2_ and PLY. The major source of H_2_O_2_ generated by *S. pneumoniae* is pyruvate oxidase (SpxB), which converts pyruvate to acetyl phosphate, CO_2_, and H_2_O_2_ [14,15,32]. Apart from SpxB, lactate oxidase (LctO) also contributes to endogenous H_2_O_2_ production, albeit to a lesser extent, by catalyzing the conversion of L-lactate to pyruvate and H_2_O_2_ [14]. Although the generation of high (up to millimolar) concentrations of H_2_O_2_ as a by-product of SpxB and LctO is known to be of great importance for *S. pneumoniae* biology, it has not been determined whether H_2_O_2_ affects the activity of PLY. 

**Figure 1:**
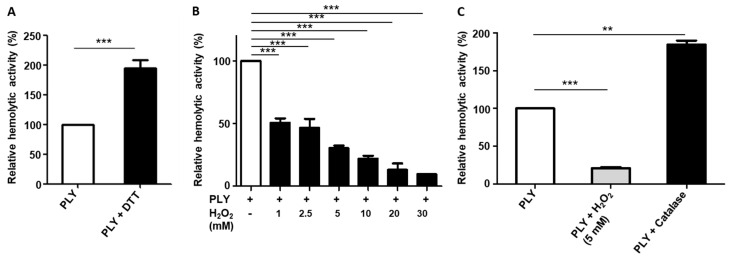
DTT and H_2_O_2_ affect the haemolytic activity of PLY. The haemolytic activity of PLY was measured in the presence of 1% sheep erythrocytes at 37 °C for 1 h. Results are shown as relative haemolytic activity. (**A**) PLY haemolytic activity is increased in the presence of DTT (5 mM). (**B**) Reduced haemolytic activity of PLY upon treatment with different H_2_O_2_ concentrations (1, 2.5, 5, 10, 20, and 30 mM) for 20 min. (**C**) Enhancement of the haemolytic activity of PLY in the presence of catalase (1000 U/mL). Significant differences between groups are denoted with asterisks (*p* < 0.01 = **; *p* < 0.001 = ***).

As shown in Figure 1A, the reducing agent dithiothreitol (DTT), which is known to prevent and reverse intra- and intermolecular disulfide bond formation between cysteine residues [33], significantly enhances the haemolytic activity of purified PLY, the major pathogenic factor of *S. pneumoniae*. Since PLY lacks a secretion signal sequence, its release does not occur actively [6,7]. Hence, the toxin is exposed to high endogenous amounts of H_2_O_2_ within *S. pneumoniae* cells. We wondered if H_2_O_2_-induced cysteine modifications affect the activity of PLY. We found that pre-incubation of purified PLY with different H_2_O_2_ concentrations (1, 2.5, 5, 10, 20, and 30 mM) reduced the haemolytic activity of PLY in a concentration-dependent manner (Figure 1B). In contrast, the presence of catalase, an H_2_O_2_-degrading enzyme, almost doubled the haemolytic activity of PLY to 183%, as compared to that of untreated PLY (Figure 1C). 

### 2.2. The Unique Cysteine Residue of PLY Contributes to the H_2_O_2_-Dependent Decrease in Its Haemolytic Activity

**Figure 2:**
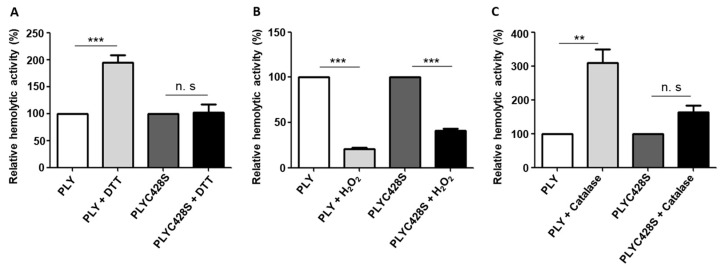
Substitution of the unique cysteine residue in PLY impairs the effects of DTT and H_2_O_2_ on the haemolytic activity of PLY. (**A**) Pre-treatment of PLYC428S with the reducing agent DTT (5 mM) does not enhance its haemolytic activity, in contrast to wild-type PLY. (**B**) H_2_O_2_ treatment [(5 mM) for 20 min] reduces the haemolytic activity of wild-type PLY more than that of PLYC428S. (**C**) Treatment of wild-type PLY and PLYC428S with catalase (1000 U/mL). The positive effect of catalase on the haemolytic activity of PLY is reduced in the absence of the unique cysteine residue. The haemolytic activity of PLY was measured in the presence of 1% sheep erythrocytes at 37 °C for 1 h. The results are shown as relative haemolytic activity. Significant differences are denoted with asterisks (*p* < 0.01 = **; *p* < 0.001 = ***). n. s. = not significant.

We investigated whether the H_2_O_2_-induced changes in the haemolytic activity of PLY could be due to H_2_O_2_-triggered sulfenylation of the unique and highly conserved cysteine residue (aa 428), which is localized in the undecapeptide sequence of PLY. This cysteine residue is required for membrane binding and is highly conserved among members of the CDC family [34]. Generally, cysteine residues are susceptible to oxidation by reactive oxygen species (ROS) because they have a highly nucleophilic nature [35]. Oxidation of thiol groups to sulfenic acid (R-SOH) is a reversible modification of cysteine-containing proteins [36]. In order to investigate whether H_2_O_2_-related sulfenylation alters the haemolytic activity of PLY, we substituted the unique cysteine residue (aa 428) of the toxin for a serine residue (PLYC428S). In contrast to wild-type PLY, pre-treatment of PLYC428S with the reducing agent DTT did not increase its haemolytic activity (Figure 2A). This result suggests that cysteine modifications in PLY are involved in the DTT-triggered increase in haemolytic activity. In addition, the reduction in PLY haemolytic activity following pre-treatment with H_2_O_2_ was significantly smaller in mutant PLYC428S (58%) than in wild-type PLY (79%) (Figure 2B). Furthermore, catalase had a significantly larger impact on the haemolytic activity of wild-type PLY (309%—Figure 1C) than on that of PLYC428S (164%) (Figure 2C). 

### 2.3. Supernatants of S. pneumoniae Mutant Strains with Impaired H_2_O_2_ Production Have Reduced Haemolytic Activity

**Figure 3:**
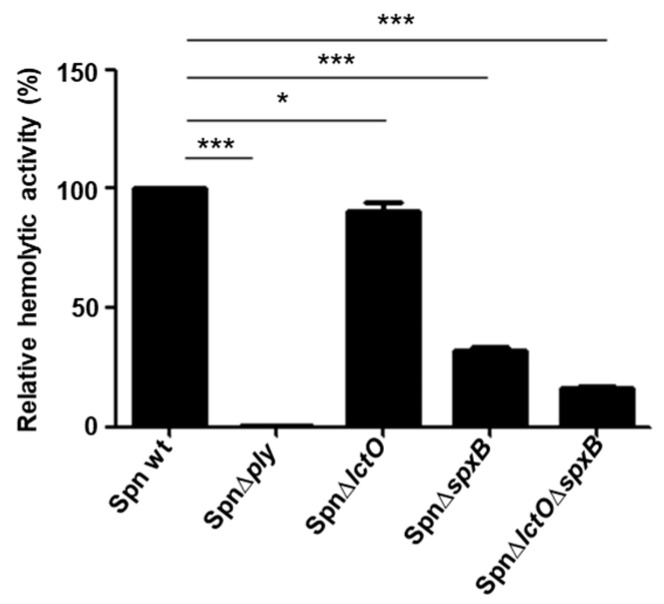
Supernatants of the *S. pneumoniae* mutant strains Spn∆*lctO*, Spn∆*spxB*, and Spn∆*lctO*∆*spxB* have decreased haemolytic activities. The relative haemolytic activities of concentrated supernatants of the three mutants are decreased in comparison to the wild type. The PLY deletion mutant (∆*ply*) exhibits no haemolytic activity due to the absence of PLY. Significant differences between groups are denoted with asterisks (*p* < 0.05 = *; *p* < 0.001 = ***).

As the pre-incubation of purified PLY with H_2_O_2_ decreases the activity of PLY, we wanted to figure out whether endogenously produced pneumococcal H_2_O_2_ affects PLY activity. Therefore, we determined the haemolytic activities of PLY-containing supernatants from *S. pneumoniae* strains that are impaired in H_2_O_2_ production. Since SpxB is considered the major source of H_2_O_2_ production, pneumococcal mutants lacking its expression generate only 13% of the production found in the wild-type *S. pneumoniae* strain. Likewise, a mutant strain lacking LctO produces only 38%, and a double-mutant *S. pneumoniae* strain lacking both (Δ*lctO*Δ*spxB*) generates only 3% of the H_2_O_2_ quantity found in the wild-type strain [14]. The supernatants of wild-type *S. pneumoniae*; the mutants Spn∆*lctO*, Spn∆*spxB*, and Spn∆*lctO*∆*spxB*; and the PLY deletion mutant (∆*ply*) cultures in the exponential growth phase were concentrated, and their individual haemolytic activities were determined. In contrast to our expectation that the mutant strains would release more active PLY due to their impaired H_2_O_2_ production, the results show a gradual decrease in the haemolytic activity of the mutant supernatants as compared to that of the wild-type strain (Figure 3). Intriguingly, the reduction in haemolytic activities correlated with the amounts of H_2_O_2_ produced by the tested mutants. Consequently, the most vigorous decrease in the haemolytic activity among the strains that are impaired in H_2_O_2_ production was detected in the supernatant of the double mutant SpnΔ*lctO*Δ*spxB* (83% reduction), followed by the supernatant of Spn∆*spxB* (68% decrease), and finally, a minor decrease (9%) was detected in the supernatant of Spn∆*lctO* (Figure 3). The supernatant of the PLY deletion mutant (Spn∆*ply*), which was tested as a negative control, exhibited no haemolytic activities, as expected due to the absence of PLY. 

### 2.4. Endogenously Produced Pneumococcal H_2_O_2_ Affects PLY Release

**Figure 4:**
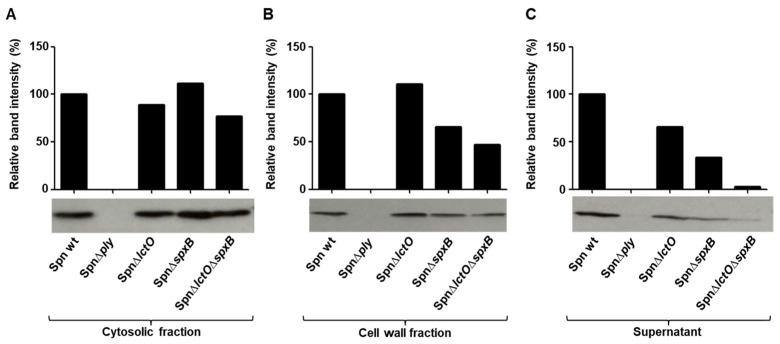
Endogenously produced H_2_O_2_ affects PLY release in *S. pneumoniae*. Detection of the PLY amounts produced by wild-type *S. pneumoniae* (Spn WT), Spn∆*ply*, Spn∆*lctO*, Spn∆*spxB*, and Spn∆*lctO*∆*spxB* in the (**A**) cytosolic fraction; (**B**) cell-wall fraction; (**C**) supernatant using a specific anti-PLY antibody. The data represent one of three biological replicates.

To investigate whether the differences in haemolytic activities in the supernatants of the mutants SpnΔ*lctO*, SpnΔ*spxB*, and SpnΔ*lctO*Δ*spxB* cultures are due to impaired PLY expression or variations in release, we first analyzed the PLY amounts in the cytosolic fractions of the wild-type strain and the three deletion mutants. As depicted in Figure 4A, using a PLY-specific antibody, the detected PLY amounts in the cytosol are comparable between the wild-type strain and the three mutants (SpnΔ*lctO*, SpnΔ*spxB*, and SpnΔ*lctO*Δ*spxB*). Expectedly, no PLY is detectable in the PLY deletion mutant (Spn∆*ply*). These results indicate that the differences in terms of haemolytic activities in the supernatants are not due to differences in PLY expression. 

In contrast to the results from the cytosolic fractions, quantification of PLY in the cell wall and especially in the supernatant fractions revealed clear differences in the cell-wall and released PLY amounts between the wild-type strain and the mutants impaired in H_2_O_2_ production (Figure 4B,C). The supernatant fractions demonstrated a clear correlation between the endogenously generated H_2_O_2_ amounts in the mutants and the quantity of released PLY, with the lowest amounts of the toxin detected in the supernatant of SpnΔ*lctO*Δ*spxB*, followed by SpnΔ*spxB* and finally SpnΔ*lctO* (Figure 4C).

### 2.5. Upregulation of Cell-Wall Synthesis and Choline-Binding Genes in S. pneumoniae Mutants Impaired in H_2_O_2_ Production

In order to obtain a better insight into the mechanisms of decreased PLY release in the supernatants of Spn∆*lctO*, Spn∆*spxB*, and Spn∆*lctO*∆*spxB* mutants, the transcriptome of the three mutants was investigated during the exponential growth phase and was compared to that of the wild-type strain. Interestingly, the absence of SpxB and LctO caused an increased expression of several genes involved in peptidoglycan synthesis and choline-binding protein (CPB) synthesis (Table S2). The group of CPBs includes cell-wall hydrolases and adhesins. CBPs consist of a functional domain and a choline-binding domain that allows them to form non-covalent bonds with choline residues in the cell wall of host cells [37]. 

**Figure 5:**
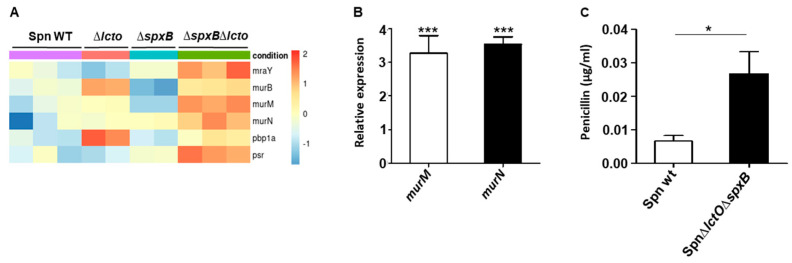
Increased transcription of cell-wall proteins in Spn∆*lctO*∆*spxB*. (**A**) Heat map of transcriptome analysis of differentially expressed genes involved in cell-wall synthesis of wild-type (Spn WT), Spn∆*lctO*, Spn∆*spxB*, and Spn∆*lctO*∆*spxB* strains. (**B**) Relative expression of *murN* and murM in Spn WT and Spn∆*lctO*∆*spxB*. (**C**) Minimal inhibitory concentration (MIC) of penicillin in Spn WT and Spn∆*lctO*∆*spxB* strains demonstrates reduced sensitivity of the double-mutant strain compared to that of the wild type. Significant differences are denoted with asterisks (*p* < 0.05 = *; *p* < 0.001 = ***).

The genes *mraY* (phospho-N-acetylmuramoyl-pentapeptide-transferase), *murB* (UDP-N-acetylmuramate dehydrogenase), *murM* (peptidoglycan bridge formation alanyltransferase), *murN* (peptidoglycan bridge formation alanyltransferase), *pbp1a* (penicillin-binding protein A), and *psr* (polyisoprenyl-teichoic acid-peptidoglycan teichoic acid transferase) involved in peptidoglycan synthesis were found to be upregulated in the Spn∆*lctO*∆*spxB* mutant strain compared to levels in the wild type (Figure 5A). As the most pronounced differences were detected in the transcriptome of the mutant Spn∆*lctO*∆*spxB* strain, we focused the following experiments on the differences between wild-type *S. pneumoniae* and this double-mutant strain. The observed variability in the expression levels of selected genes involved in peptidoglycan synthesis and the encoding of choline-binding proteins was further substantiated via quantitative real-time PCR analysis. The increased expression of the genes *murM* and *murN* encoding the peptidoglycan bridge formation alanyltransferases in Spn∆lctO∆*spxB* could as such be confirmed (Figure 5A,B). Interestingly, MurM and MurN have been described to play a role in inhibiting PLY release through the formation of branched stem peptides in peptidoglycan [38]. In order to investigate the consequences of the increased expression of genes involved in cell-wall synthesis in the Spn∆*lctO*∆*spxB* mutant strain, we assessed its sensitivity to penicillin, which targets the cell wall. Spn∆*lctO*∆*spxB* was found to be less sensitive to penicillin than the wild type (Figure 5C).

**Figure 6:**
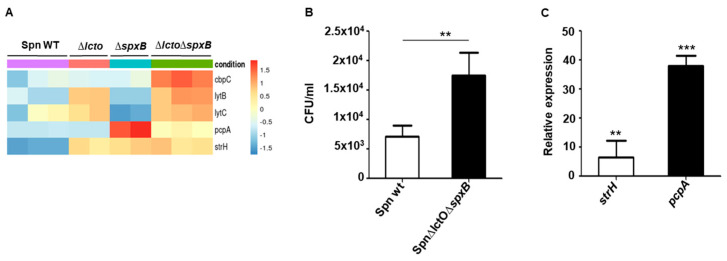
Increased transcription of choline-binding proteins correlates with increased adhesion of Spn∆*lctO*∆*spxB* in H441 lung cells. (**A**) Heat map of differentially expressed CBP-encoding genes. (**B**) Adhesion of Spn∆*lctO*∆*spxB* and Spn WT to H441 cells in 30 min. (**C**) Relative expression of *strH* (LPXTG-anchored beta-N-acetylhexosaminidase) and *pcpA* (choline-binding protein A) in wild-type *S. pneumoniae* and Spn∆*lctO*∆*spxB*. Significant differences are denoted with asterisks (*p* < 0.01 = **; *p* < 0.001 = ***).

The CPBs encoding genes *cbpC* (choline-binding protein), *lytB* (endo-beta-N-acetylglucosaminidase), and *lytC* (choline-binding-anchored murein hydrolase), as well as proteins *pcpA* (choline-binding protein A) and *strH* (LPXTG-anchored beta-N-acetylhexosaminidase), were found to be upregulated in the Spn∆*lctO*∆*spxB* mutant strain compared to levels in the wild type (Figure 6A). We investigated the adhesion behaviour of wild-type *S. pneumoniae* and Spn∆*lctO*∆*spxB* towards human lung epithelial cells (H441). The results show that within 30 min, Spn∆*lctO*∆*spxB* adheres significantly better (3-fold) to H441 cells than the wild type (Figure 6B). The upregulated choline-binding proteins PcpA and StrH were described to elevate host-cell adherence of pneumococci [39]. The significantly enhanced expression of *strH* and *pcpA* in Spn∆*lctO*∆*spxB* was confirmed via quantitative real-time PCR analysis (Figure 6C).

## 3. Discussion

*S. pneumoniae* is the leading cause of community-acquired pneumonia in the elderly and of bacterial pneumonia in children under 5 years of age worldwide [40]. Pneumococcal infection induces severe invasive disease. The pathogen releases two major virulence factors, H*_2_*O*_2_* and the pore-forming toxin pneumolysin (PLY). H_2_O_2_ is a by-product of the metabolism of the enzymes pyruvate oxidase (SpxB) and lactate oxidase LctO [14,15]. Both virulence factors induce overlapping stress responses in host epithelial and endothelial cells, including production of mitochondrial reactive oxygen species (mtROS), re-localization of host-cell chaperones, modification of the cytoskeleton, loss of apically located ion channels, and barrier disruption [12,23,30,41]. As PLY lacks an *N*-terminal secretion signal sequence, the toxin is not actively released and remains stored in the cytoplasm [6,7]. The missing active release could extend the time for which PLY is exposed to high concentrations of endogenously generated H_2_O_2_ by *S. pneumoniae*. It has been shown that *S. pneumoniae* exploits endogenous H_2_O_2_ as an intracellular signalling molecule that modulates the activity of several proteins via protein sulfenylation [14]. However, so far it has not been clarified whether H_2_O_2_ affects the activity of PLY. Our results demonstrate that H_2_O_2_ reduces the haemolytic activity of PLY in a concentration-dependent manner. In correlation with these findings, the degradation of H_2_O_2_ upon the addition of catalase significantly increases the haemolytic activity of PLY. The improved haemolytic activity of PLY in the presence of catalase is probably achieved by degradation of self-generated H_2_O_2_ in erythrocytes, which are applied to determine the haemolytic activity of PLY. Auto-oxidation of haemoglobin to methaemoglobin in erythrocytes generates superoxide anions, which can be catalyzed to produce H_2_O_2_ [42]. We propose that the H_2_O_2_-induced reduction in PLY activity is due to sulfenylation of the unique and highly conserved cysteine residue in position 428, located in the undecapeptide sequence that is necessary for host-cell membrane binding [34]. Indeed, sulfenylation is a reversible modification of the thiol groups in cysteine residues to sulfenic acid (R-SOH), which can be carried out by H_2_O_2_ [36]. The substitution of cysteine to serine in PLYC428S abolished the ability of DTT to activate PLY. The unresponsiveness of PLYC428S to the thiol reducing reagent DTT indicates that the effect of DTT on PLY depends exclusively on the presence of the unique cysteine residue in PLY. In addition, the absence of cysteine reduced the susceptibility of modified PLY to H_2_O_2_ treatment about two-fold compared to that of wild-type PLY, which might be attributed to the prevention of sulfenylation. Although catalase treatment enhanced the haemolytic activity of PLYC428S (by 164%), this increase was only about half of that measured for wild-type PLY (309%). The observed ability of H_2_O_2_ to affect the haemolytic activity of PLY prompted us to investigate PLY activity in strains that are impaired in H_2_O_2_ generation. As such, we found a clear correlation between the haemolytic activity of the tested strains and their capability to generate H_2_O_2_. Contrary to our expectations, the largest decrease in haemolytic activity was detected in the supernatant of the double mutant SpnΔ*lctO*Δ*spxB*, followed by Spn∆*spxB* and finally Spn∆*lctO*. This order correlates exactly with the generated H_2_O_2_ amounts in the mutants, with SpnΔ*lctO*Δ*spxB* producing only 3% of control, Spn∆*spxB* 13%, and Spn∆*lctO* 38% [14]. The significantly reduced amounts of extracellular PLY detected in the supernatants of the mutant strains with impaired H_2_O_2_ generation compared to the wild type provides a plausible explanation for the detected differences in haemolytic activities of the tested strains. The results show a clear gradual correlation between endogenous H_2_O_2_ generation and PLY release. The less H_2_O_2_ is produced, the less PLY is released, despite similar PLY expression levels in the cytosol of the tested strains. Bryant and co-workers have reported that H_2_O_2_ generated by SpxB contributes to PLY release in clinical *S. pneumoniae* isolates and that addition of exogenous H_2_O_2_ did not change this. In their study, catalase supplementation prevented PLY release in some strains, which indicates that intracellularly generated rather than exogenous added H_2_O_2_ promotes PLY release in *S. pneumoniae* [43]. Additionally, deoxycholate-induced autolysis of *S. pneumoniae* was significantly reduced in strains that lack SpxB, indicating a possible impairment of the cell membrane when SpxB is expressed [43]. Furthermore, SpxB-derived H_2_O_2_ in *S. pneumoniae* inactivates FabF via oxidation of the cysteine thiol residue in the active site and thereby alters fatty acid composition in the membrane [44]. 

We applied RNA-Seq to provide insight into the reasons for the influence of endogenously generated H_2_O_2_ on PLY release. The comparison of expression profiles of wild-type *S. pneumoniae* and the three mutants Spn∆*lctO*, Spn∆*spxB*, and Spn∆*lctO*∆*spxB* revealed that reduced endogenous levels of H_2_O_2_ caused by the absence of SpxB and LctO caused enhanced expression of several genes involved in peptidoglycan synthesis. Before release, PLY localizes in the cell wall of growing *S. pneumoniae.* It has been shown that the peptidoglycan structure restrains the PLY release into the extracellular environment [38]. The peptidoglycan bridge formation alanyltransferases MurM and MurN are involved in the formation of branched stem peptides in peptidoglycan, the formation of which inhibits PLY release [38]. Our transcriptome analysis showed that the genes encoding *murM* and *murN* were upregulated in the double mutant SpnΔ*lctO*Δ*spxB*, and this was validated by RT-PCR. Furthermore, it has been shown that *murMN* expression is necessary for penicillin resistance [45]. The assessment of penicillin sensitivity in wild-type *S. pneumoniae* and SpnΔ*lctO*Δ*spxB* indicated increased resistance of the double mutant against penicillin. These findings suggest a contribution of the increased expression of *murN* and *murM* in Spn∆*lctO*∆*spxB* to decreased PLY release and enhanced resistance against penicillin, possibly in combination with additional factors involved in peptidoglycan synthesis and increased CBP expression. On the other hand, the transcriptome analysis unveiled the upregulation of several genes encoding CBPs in Spn∆*lctO*∆*spxB*. The cell-surface protein PcpA (encoding choline-binding protein A) mediates the adherence of *S. pneumoniae* in the human nasopharynx and to epithelial lung cells and is present in clinically relevant *S. pneumoniae* strains [46,47]. Anti-pcpA antibody from human serum reduces the adherence of pneumococci [46]. In a mouse carriage model, it was shown that PcpA plays a role in the colonization of the lungs [48]. StrH is an exo-β-D-N-acetyl-glucosaminidase involved in processing of *N*-linked glycans of human glycoconjugates and host–bacteria interactions [49]. It is implicated in the colonization of the airways [50]. In accordance with the enhanced expression of genes involved encoding CBPs, we found elevated adherence of Spn∆*lctO*∆*spxB* mutants to human lung epithelial cells (H441) compared to the wild-type strain. The expression of the hydrolases *lytB* and *lytC* was also found to be upregulated in Spn∆*lctO*∆*spxB* as compared to the wild type. It has been shown in vitro and in vivo that LytB and LytC have a role in the attachment of *S. pneumoniae* to human nasopharyngeal cells [51]. Therefore, the upregulation of *lytB* and *lytC* in Spn∆*lctO*∆*spxB* might also contribute to the increased ability of Spn∆*lctO*∆*spxB* to adhere to epithelial lung cells.

We presume that the enhanced expression of genes involved in the production of CPBs and in PG synthesis is rather a compensatory effect for the reduced H_2_O_2_ caused by the absence of SpxB and LctO levels in the mutant strains. The expression is strikingly increased in the double mutant (Spn∆*lctO*∆*spxB*). The individual deletion of *lctO* or *spxB* generally shows a lower increase (Figure 5A and Figure 6A). However, indirect effects due to metabolic changes cannot be excluded. The deletion of *spxB* and *lctO* affects the pyruvate node, which is an important branch within the central carbon metabolism in *S. pneumoniae* and acts as a connection point between glycolysis, gluconeogenesis, and the TCA cycle. Possible consequences of such metabolic variations on the expression levels of choline-binding protein and PG remain to be elucidated.

## 4. Conclusions

In summary, we found that H_2_O_2_ reduces the haemolytic activity of PLY. This activity depends at least partially on the unique and highly conserved cysteine residue in PLY. We substantiated these findings by demonstrating a clear gradual correlation between endogenous H_2_O_2_ generation and PLY release in wild-type *S. pneumoniae*, Spn∆*lctO*, Spn∆*spxB*, and finally Spn∆*lctO*∆*spxB*, the last of which produces the smallest amounts of H_2_O_2_ and releases the lowest levels of PLY. Transcriptome analyses suppose changes in the peptidoglycan composition and CBP production as a result of reduced endogenous levels of H_2_O_2_. Further research is needed to elucidate the exact structural modifications in the peptidoglycan and the cell envelope of *S. pneumoniae* that arise as a consequence of reduced H_2_O_2_ generation.

## 5. Materials and Methods

### 5.1. Bacterial Strains and Growth Conditions

Wild-type *S. pneumoniae* D39, Spn∆*ply* (kindly provided by Prof. Hammerschmidt (University Greifswald, Germany)) and the deletion mutants Spn∆*lctO*, Spn∆*spxB*, and Spn∆*lctO*∆*spxB* (kindly provided by Prof. Winkler and Prof. Giedroc (University of Indiana, USA) were freshly plated from cryocultures and stored at −80 °C on blood agar plates. Then the strains were incubated overnight for 15–17 h at 33 °C/5% CO_2_. The grown colonies were inoculated into Todd–Hewitt broth medium (BD) containing 0.5% yeast extract (THB-Y) to an OD_600 nm_ of 0.04 and incubated at 37 °C/5% CO_2_.

### 5.2. Generation of PLYC428S and Isolation of PLY 

The PLY encoding sequence was synthesized (Genscript, Piscataway, NJ, USA) and modified to substitute the cysteine codon by serine. LPS-free wild-type PLY and PLYC428S were purified from a recombinant *Listeria innocua* 6a strain [52].

### 5.3. Haemolysis Assay

The haemolytic activity of purified PLY and pneumococcal supernatants was determined using human erythrocytes washed in phosphate-buffered saline (pH 7.4) as described previously [53].

### 5.4. Detection and Quantification of PLY

For the detection of PLY in cytosol, cell wall, and supernatant fractions, *S. pneumoniae* strains were cultured until OD_600 nm_ 1.0 as described in Section 5.1. The grown cultures were centrifuged at 6000 rpm for 10 min. The supernatants were sterile filtered (0.22 µm Millex-GV filter, Merck Millipore Burlington, Burlington, MA, USA), concentrated using Amicon Ultra–4 centrifugal filters (Merck Millipore Burlington, Burlington, MA, USA), and then used for detection of PLY. The bacterial pellets were lysed in cell-wall digestion buffer [54] and incubated at 350 rpm for 3 h. The cell-wall and cytosolic fractions were separated by centrifugation for 10 min at 14,100× *g* at 4 °C. Protein concentrations were determined using the Bradford assay (Bio-Rad Laboratories, Hercules, CA, USA). Equal amounts of protein from each sample were loaded onto SDS-PAGE and transferred to PVDF membranes (Hoffman-La Roche AG, Basel, Schweiz). After blotting, the membranes were blocked using 5% milk (powdered milk, Carl Roth GmbH + Co. KG, Karlsruhe, Germany), solved in Tris-buffered saline with Tween20 (SERVA Electrophoresis, Heidelberg, Germany), and incubated with specific mouse anti-PLY monoclonal antibody (sc-80500, Santa Cruz Biotechnology Inc, Dallas, TX, USA). Subsequently, the samples were treated with horseradish peroxidase-conjugated goat anti-mouse antibody (sc-516102, Santa Cruz Biotechnology Inc, Dallas, TX, USA). The protein bands were visualized using an ECL detection reagent. The quantification of the band intensities was performed using Software Fiji Image J (ImageJ2).

### 5.5. Minimum Inhibitory Concentration (MIC) Determination

Serial dilutions of penicillin (100 µL) solved in THB-Y medium were distributed into wells of 96-well microtiter plate (Thermo Fisher Scientific Inc., Waltham, MA, USA). Subsequently, 100 µL of the bacterial suspension (10^7^ CFU/mL) was added into the wells containing the penicillin dilutions. The microtiter plate was incubated at 37 °C/5% CO_2_ for 18 to 24 h. The MIC was defined as the lowest antibiotic concentration at which no visible growth could be detected.

### 5.6. Cell Culture Conditions

The human lung adenocarcinoma cell line NCI-H441 (H441) was cultured in Roswell Park Memorial Institute (RPMI) 1640 medium (Gibco by Life Technologies, Carlsbad, CA, USA) with 10% heat-inactivated foetal calf serum (FCS) (Superior, Thermo Fisher Scientific Inc., Waltham, MA, USA) at 37 °C/5% CO_2_. 

### 5.7. Adhesion Assays

H441 cells were seeded in 12-well plates at a density of 5×10^5^ cells/well and incubated at 37 °C/5% CO_2_ for 24 h before conducting the assay. *S. pneumoniae* cultures grown for 3 h were washed 2× with PBS and resuspended in RPMI medium supplemented with 10% FCS. Wild-type *S. pneumoniae* D39 or Spn∆*lctO*∆*spxB* were added to H441 cells for 30 min. Then, the cell culture supernatants were removed, and the remaining H441 cells were washed 5 times using PBS. Finally, the cells were trypsinized using 200 µL Trypsin (0.05% Trypsin/0.02% EDTA without Calcium; Bio&Sell GmbH, Feucht, Germany). The detached cell suspensions were plated on brain–heart infusion (BHI) agar plates and incubated at 37 °C/5% CO_2_ to determine the CFU.

### 5.8. RNA Isolation

*S. pneumoniae* cultures (OD_600 nm_ 1.0) were aliquoted to 0.5 mL and treated with 1.0 mL RNA protect (Qiagen N.V, Venlo, The Netherlands) for 5 min at RT. Bacterial cells were then collected by centrifugation at 13,000 rpm for 5 min and stored at −80 °C. RNA was isolated using a modified protocol of the miRNAse kit (Qiagen N.V, Venlo, The Netherlands) according to Mraheil et al. 2011 [55]. Shortly thereafter, the collected bacterial pellets were thawed and washed with SET buffer [50 mM NaCl, 5 mM EDTA, and 30 mM Tris-HCl (pH 7.0)]. Subsequently, cells were centrifuged at 16,000× *g* for 3 min, and pellets were resuspended in Tris-HCl (pH 6.5) containing 40 U of SUPERase, 0.2 mg of proteinase K (Ambion, Kaufungen, Germany), 50 mg/mL lysozyme (Sigma-Aldrich, St. Louis, MO, USA), and 25 U of mutanolysin (Sigma-Aldrich, St. Louis, MO, USA). After incubation for 30 min at 37 °C and 350 rpm, QIAzole (Qiagen N.V, Venlo, The Netherlands) was added for 3 min at RT. Then, 0.2 volume chloroform was added followed by incubation for 3 min at RT. After centrifugation (16,000× *g* at 4 °C for 15 min), the upper aqueous phase containing the RNA was transferred to a new collection tube. Next, 1.5-volume ethanol (100%) was added to the sample and mixed. The aqueous phase and ethanol were transferred to miRNeasy Kit column (Qiagen N.V, Venlo, The Netherlands) and treated according to the user’s manual. 

### 5.9. Quantitative RT-PCR 

Quantitative RT-PCR was performed according to Mraheil et al. 2011 [55]. After RNA isolation, RNA (500 ng) was treated with DNase (RNase-free DNase Set, Qiagen N.V, Venlo, The Netherlands), and cDNA was generated with SuperScript II reverse transcriptase (Invitrogen AG, Carlsbad, CA, USA). Amplification of cDNA samples was performed using the QuantiTect SYBR Green PCR Kit (Qiagen N.V, Venlo, The Netherlands) and the StepOnePlus RT-PCR System (software V2.2.2)(Applied Biosystems by Life Technologies, Carlsbad, CA, USA)Primers for genes amplified with real-time PCR are shown in Table S1, including primers for reference gene 16S rRNA. Gene expression levels were calculated using the mathematical model for relative quantification in real-time PCR according to Pfaffl, 2001 [56]. 

### 5.10. Library Preparation for RNA-Sequencing

The concentration of total RNA was measured with an Invitrogen™ Qubit™ 3 Fluorometer and a Qubit™ RNA High Sensitivity (HS) Kit (Thermo Fisher Scientific Inc., Waltham, MA, USA). Quality was assessed using the Bioanalyzer 2100 instrument with an RNA Nano 6000 Kit (Agilent, Santa Clara, CA, USA). Next, total RNA was depleted of ribosomal RNA with a NEBNext^®^ rRNA Depletion Kit v2 (Bacteria) (New England Biolabs, Ipswich, MA, USA) and Agencourt RNAClean XP beads (Beckman Coulter, Pasadena, CA, USA). Fragmentation and library preparation was then performed according to the manufacturer’s instructions using an NEBNext^®^ Ultra II Directional RNA Library Prep Kit for Illumina. To facilitate multiplex sequencing, NEBNext^®^ Multiplex Oligos for Illumina^®^ (96 Unique Dual Index Primer Pairs) (New England Biolabs, Ipswich, MA, USA) was used to barcode the reads. Libraries were further purified with Agencourt AMPure XP Reagent (Beckman Coulter, Pasadena, CA, USA), and the quality and quantity of the amplified cDNA were assessed with a DNA High Sensitivity Kit (Agilent, Santa Clara, CA, USA). Following this, they were pooled, denatured, and diluted to 1.8 nM. Single-end reads were sequenced with NextSeq 500/550 Mid Output Kit v2.5 (150 cycles) reagents (Illumina, San Diego, CA, USA). 

### 5.11. RNA-Seq Analyses

Before analysis, the sequenced reads were image-processed, basecalled, and demultiplexed. All reads obtained were aligned to *Streptococcus pneumoniae* strain D39 (NCBI GCF_023635025.1_ASM2363502v1) using STAR [57]. Aligned reads were sorted by position using samtools [58], and read counts per gene or transcript were calculated for each library using the subread function featureCounts [59]. Differential gene expression analyses were performed with the R-package DESeq2 [60]. Outliers were identified and removed from the downstream analysis. The data was visualized using several packages available for R, namely pheatmap [61], ggplot2 [62], and reshape2 [63] for heatmaps; ggrepel [64], EnhancedVolcano [65], and RColorBrewer [66] for volcano plots; and ggvenn [67] and gplots [68] for Venn diagrams. For data manipulation, the R tools tidyverse [69], forcats [70], and dplyr [71] were used, as well as openxlsx [72] to export tables. Data were normalized per ‘regularized log’ transformation and shrunk using the adaptive shrinkage estimator for the ashr package [73]. Genes were considered differentially expressed when the adjusted *p*-value was below 0.05 and the logarithmized fold change was above 1 or below −1.

### 5.12. Statistical Analyses

Statistical analyses were carried out using one-way ANOVA, unpaired *t*-tests, and Mann–Whitney tests. All analyses were performed using GraphPad Prism software 5 (GraphPad Software, Inc., La Jolla, CA, USA). *p* < 0.05 was considered to indicate a statistically significant difference.

## Data Availability

The data presented in this study are available on request from the corresponding author.

## Supplementary Materials

The following supporting information can be downloaded at: https://www.mdpi.com/article/10.3390/toxins15100593/s1, https://www.ncbi.nlm.nih.gov/geo/query/acc.cgi?acc=GSE240442 (accessed on 9 August 2023); Table S1: Primers used for RT-PCR; Table S2: Comparative transcriptome sequencing analysis of the wild-type *S. pneumoniae* strain and three mutants impaired in H_2_O_2_ production.
