# Supplementary material for: Impact of Endogenous Pneumococcal Hydrogen Peroxide on the Activity and Release of Pneumolysin
Source: Toxins (Basel). 2023 Sep 30;15(10):593. doi: 10.3390/toxins15100593 (PMC10611280; doi:10.3390/toxins15100593)
Supplement: Supplementary file 1 [file toxins-15-00593-s001.zip › Table S1.pdf]

Table S1. Primers used for RT PCR

Primer	Sequence (5`-3`)
16S rRNA-fw	GGTGAGTAACGCGTAGGTAA
16S rRNA-rev	ACGATCCGAAAACCTTCTTC
murN-fw	CAAGCGGAGTTTCTCATAGC
murN-rev	CAAGTCTCGCGCTTCTG
murM-fw	CTATGCTCGCAGTAAGAGAGTG
murM-rev	CACCTTACTCCCATTTGTTGC
pcpA-fw	CTTGACCAGCTTCAAACTCTAC
pcpA-rev	CAGCTGCGGTTATTTTAGCAG
strH-fw	GTCCTGCGACTACTAGTTCTG
strH-rev	GTCAATGGACATCGTAGCAAC
